# Statistical genetics and polygenic risk score for precision medicine

**DOI:** 10.1186/s41232-021-00172-9

**Published:** 2021-06-17

**Authors:** Takahiro Konuma, Yukinori Okada

**Affiliations:** 1 Department of Statistical Genetics, Osaka University Graduate School of Medicine, 2-2 Yamadaoka, Suita, 565-0871 Japan; 2 Central Pharmaceutical Research Institute, Japan Tobacco Inc., Takatsuki, 569-1125 Japan; 3 Laboratory of Statistical Immunology, Immunology Frontier Research Center (WPI-IFReC), Osaka University, Suita, 565-0871 Japan; 4 Integrated Frontier Research for Medical Science Division, Institute for Open and Transdisciplinary Research Initiatives, Osaka University, Suita, 565-0871 Japan

**Keywords:** Statistical genomics, Genome-wide association study, Polygenic risk score, Precision medicine

## Abstract

The prediction of disease risks is an essential part of personalized medicine, which includes early disease detection, prevention, and intervention. The polygenic risk score (PRS) has become the standard for quantifying genetic liability in predicting disease risks. PRS utilizes single-nucleotide polymorphisms (SNPs) with genetic risks elucidated by genome-wide association studies (GWASs) and is calculated as weighted sum scores of these SNPs with genetic risks using their effect sizes from GWASs as their weights. The utilities of PRS have been explored in many common diseases, such as cancer, coronary artery disease, obesity, and diabetes, and in various non-disease traits, such as clinical biomarkers. These applications demonstrated that PRS could identify a high-risk subgroup of these diseases as a predictive biomarker and provide information on modifiable risk factors driving health outcomes. On the other hand, there are several limitations to implementing PRSs in clinical practice, such as biased sensitivity for the ethnic background of PRS calculation and geographical differences even in the same population groups. Also, it remains unclear which method is the most suitable for the prediction with high accuracy among numerous PRS methods developed so far. Although further improvements of its comprehensiveness and generalizability will be needed for its clinical implementation in the future, PRS will be a powerful tool for therapeutic interventions and lifestyle recommendations in a wide range of diseases. Thus, it may ultimately improve the health of an entire population in the future.

## Background

**Fig. 1:**
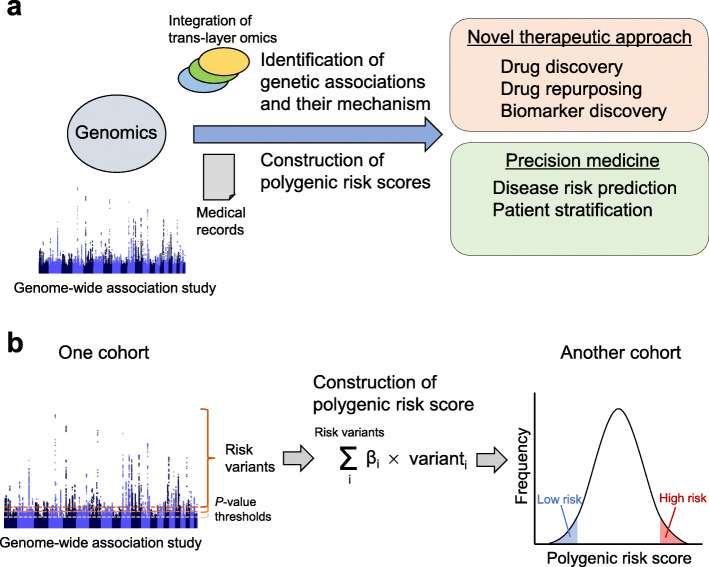
Overview of clinical application of statistical genomics and polygenic risk score. **a** Clinical application of statistical genomics for complex human diseases. **b** Overview of polygenic risk score construction. As a usual practice, PRS is calculated as a weighted sum of several risk variants from a genome-wide association study in one cohort with multiple *p*-value thresholds. The effect sizes are typically estimated as β (beta coefficients) or as odds ratios. After the PRS is calculated in one cohort, the distribution of individual PRS is assessed in another cohort

Understanding human disease risk factors that contribute to disease onset is vital for the implementation of early disease detection, prevention, and intervention. The primary components of human disease risk factors are usually explained by the combination of genetic susceptibility, environmental exposures, and lifestyle factors [1]. Differences in these factors between individuals also yield differences in disease physiology among individuals. Precision medicine can be defined as tailored medical care primarily based on understanding these differences in disease physiology among individuals (Fig. 1a).

One of the important approaches for precision medicine is stratifying individual genetic susceptibility based on inherited DNA variation. This approach has been developed with progress in human genetics. Since the first complete human genome sequencing was finished in 2003, progress in human genetics has been accelerated by recent technological advances, such as genome sequencing technology for a large population and advances in statistical genetics methodology. All this progress in human genetics has been expected to give insight into the contribution of genetic factors for common human diseases and better prediction of disease risks. A genome-wide association study (GWAS), which uses single-nucleotide polymorphisms (SNPs) arrays, is one of the most effective methods for statistically assessing the genetic association of diseases. Not only have GWASs identified thousands of genomic loci associated with common human diseases [2], they have also elucidated complex genetic architectures in most common human diseases. Because most SNPs identified by GWASs that are significantly associated with common human diseases often have a small effect size on these disease risks [3], using only SNPs that are significantly associated with these diseases in disease risk prediction is not reliable. A model underlying this concept is called a “polygenic model” [4], which explains disease susceptibility as a combination of several SNPs that have a small effect size on disease.

A statistical method based on a polygenic model, called the PRS, has been developed. PRS is calculated as a weighted sum of several risk alleles carried by an individual, where the risk alleles and their weights are defined by SNPs and their measured effects [5]. PRS has gained interest because it may be useful in predicting individual disease susceptibility. In this review, we summarize the recent advances, utilities, perspectives, and precision medicine application of PRS (Fig. 1).

## An overview of polygenic risk score

The concept of polygenic risk was initially advocated and modeled in the early twentieth century [6]. Along with the accumulation of genomic loci associated with common human diseases and complex traits by the success of GWASs [2], it has been possible to quantify polygenic risk using risk alleles identified by GWASs in these diseases and traits. The quantifying polygenic risk methods have been developed in the last decade as tools to calculate the cumulative effect of many genetic loci for a certain trait into a quantitative metric [7], which is called PRS. As a usual practice, PRS is calculated as a weighted sum of several risk alleles carried by an individual. The risk alleles and their weights are defined by SNPs and their measured effect sizes (Fig. 1b). The effect sizes are typically estimated as the beta coefficients for quantitative traits or as odds ratios for categorical binary traits. PRS is typically calculated using a set of SNPs with different *p*-value thresholds (e.g., 1 × 10^−5^, 1 × 10^−4^, …, 0.05, 0.1, …, 0.5) for disease association, and then a series of PRSs is calculated for a disease or a trait. After the PRS has been calculated in one cohort, it is essential to assess its predictive performance in another external cohort, which is not used for the construction of PRS. This performance of constructed PRS is often evaluated by the area under the receiver operating characteristic curve, called area under the curve (AUC) of a PRS, which provides a quantitative measure for the discrimination ability of a PRS [8].

In the calculation and validation of PRS, some methodological concerns have been argued [9, 10]. For example, although the construction of PRS by inclusion of larger numbers of SNPs (including SNPs that do not meet genome-wide significance) can have more predictive accuracy, it is argued whether the inclusion of those SNPs with close to zero effects in PRSs makes sense [11]. In another example, linkage disequilibrium (LD), the correlations between nearby SNPs, which leads to over-representation of high LD regions in calculating PRS, potentially reduces the predictive performance of PRS [12]. To mitigate the effect of LD, LD pruning (randomly removing one SNP from a pair in high LD), LD clumping (pruning by LD, while referentially retaining more significantly associated SNPs), or more complex methods that explicitly account for LD [13] have been used.

## Applications of the polygenic risk score for disease prediction

In the case of disease with age-dependent prevalence, such as lifestyle-related diseases, it is effective to identify the population with a high risk of disease onset in advance and implement a preventive intervention. One of the PRS utilities with high clinical values can be a predictive biomarker of disease risk. This utility of PRS has been explored in many common diseases, such as cancer, coronary artery disease, obesity, and diabetes [14–16]. For example, in coronary artery disease, PRS, which was developed by a GWAS of coronary artery disease from a dataset (validation dataset) of UK Biobank participants and applied to another dataset (test dataset) of UK Biobank participants, demonstrated that participants in the top 0.5 percentile of PRS in the test dataset had a fivefold increase in the prevalence of coronary artery disease [16]. This result showed that PRS developed by a large-scale GWAS potentially enabled the accurate prediction of disease prevalence.

In another example, disease risk prediction of breast cancer, which had been estimated from two genes, *BRCA1* and *BRCA2* [17], was expanded by the application of PRS. PRS of breast cancer based on 303 genetic variants from a GWAS of breast cancer demonstrated that women in the top 1 percentile of PRS had a fourfold increased risk of developing estrogen receptor-positive breast cancer and a sixfold decreased risk for women in the lowest 1 percentile of PRS [18]. Although this PRS had a modest AUC of 0.63, this study showed that breast cancer PRS potentially captured sufficient information to identify a high-risk subgroup of women who could be offered preventive interventions.

Application of PRS for a non-disease trait was also reported. This analysis developed PRSs of trans-biobank (BioBank Japan, UK Biobank, and FinnGen; n_total_ = 675,898) analysis of the association of several clinical biomarkers and revealed the association between high systolic blood pressure PRS and a shorter lifespan in trans-ethnic individuals and the association between obesity PRS and lifespan in Japanese and European individuals [19]. These results showed the potential application of PRS in improving population health by providing information on modifiable risk factors driving health outcomes.

From these examples, the utilities of PRS have been expected to be potential predictors of future risks of disease or health outcomes. Thus, they are expected for target treatment application, alteration of screening paradigms, and modification of non-genetic factors related to predicted high-risk phenotypes.

## Limitations and challenges for the application of polygenic risk score

We focus on several limitations on the implementation of PRSs in clinical practice. First, PRS is highly sensitive to ethnic background. The variability of PRS among ethnic groups can be explained by the differences in allele frequency, LD, and effect sizes of variants among ethnic groups [20]. Therefore, the performance of PRS drops if PRS developed from one ethnic group is applied to another ethnic group [21]. To overcome these ethnic group-specific biases, several methods have been proposed. For example, the ancestry deconvolution PRS method with consideration for an admixture of ancestry-specific partial sequence in individual genome demonstrated improved susceptibility predictions of PRS for four traits (type 2 diabetes, breast cancer, height, and body mass index [BMI]) [22]. In addition to the further development of the PRS method, future GWASs would be needed to include subjects from diverse ethnic backgrounds to improve the generalizability and utility of PRS for all populations because the majority of GWASs have been performed in European-Caucasian populations [23, 24]. In order to enlarge the benefit of PRS in non-European-Caucasian populations, whose amount of genomic data is limited compared with European-Caucasian populations, it is important that vastly increasing diversity of participants is included and analyzed in genetic studies, and open data-sharing standards of these results are needed for improving the accuracy of PRS in these populations [24].

Second, the distribution of PRS even in the population group was reported to show biases according to geographical differences. For example, geographical differences in PRSs of coronary artery disease, rheumatoid arthritis, schizophrenia, waist–hip ratio, BMI, and height were detected in Finland [25]. Whether the cause of these biased distributions was the geographical difference in disease prevalence or the difference in the genetic background by population stratification remains unclear. In another example, considerable differences of PRSs between the non-mainlanders and mainlanders in Japan were reported [26]. In this report, PRS of BMI showed that the smaller BMI PRS was observed in non-mainlanders, although the greater BMI was observed in non-mainlanders. This difference is assumed to be a result of sudden changes in environmental factors which affected non-mainlanders’ BMI in the non-mainland that preceded the reflection of genomic structure in response to these environmental changes. From these examples, the PRS could be susceptible to population structure’s geographical distributions, even within a relatively homogeneous population.

Third, given the numerous available models of PRS, it remains unclear which method is the most suitable for predicting the risks of diseases or traits. It was reported that when categorizing the existing 15 PRS methods into three groups, which consisted of (1) simple methods that selected variants below a *p*-value limit and within a LD range, (2) complex methods that selected variants by attempting to approximate the results of a mixed-model approach, and (3) ensemble methods created by taking an average of the top five PRSs weighted by their coefficients in a cross-validated logistic regression, it was shown that the simple methods generated slightly more accurate PRSs than did the complex methods [27]. Further insight into the characterization of PRS models will be needed to evaluate and compare these predictive performances. Comparable performance metrics of these PRS models would need to be systematically evaluated. For example, the PGS Catalog [28], an open resource for PRSs that has been reported recently, enables PRS analysis in a standardized format along with consistent metadata and direct comparison between scores.

Fourth, the prospect of clinical use of PRS is associated with a wide variety of ELSI (ethical, legal, and social implications) concerns, which have been also discussed in the context of monogenic genetic results and is also present in the polygenic context [29]. One of the ELSI concerns about PRS is the relevance of findings of PRS to family members. Genetic variation is shared in families and the PRS of first-degree family members are correlated [30], but this information is not as clear as in the monogenic genetic results. Guidelines developed by professional societies would be needed for both patients and providers for prompt warning about the polygenic risk to family members. Other examples of the ELSI concerns about PRS are risk of psychosocial harms, false reassurance, and overdiagnosis and overtreatment, which are typically considered in the monogenic genetic results. Further research for whether the harms of false reassurance, overtreatment and overdiagnosis materialize would be needed.

## Conclusions

In this review, we focused on recent advances, utilities, and perspectives of PRS. The predictive accuracy of PRS will continue to be improved by more extensive and diverse cohorts to construct PRS models and improve methods for PRS derivation and application. Although further improvements of its comprehensiveness and generalizability would be needed for its clinical implementation in the future, the potential clinical impacts and benefits of the PRS have been proposed and discussed. For example, PRS-informed clinical intervention, PRS-informed disease screening, and PRS-informed life planning were proposed as the potential clinical benefits [5]. Also, individual PRS measurement only needs its genome sequence, which could be taken once at a relatively low cost, and PRS will be potentially applied for various diseases and traits. PRS will guide therapeutic interventions and lifestyle recommendations in several diseases. Thus, it might ultimately improve the health of an entire population in the future.

## Data Availability

Not applicable.

## Abbreviations

PRS: Polygenic risk score
SNP: Single nucleotide polymorphism
GWAS: Genome-wide association study
AUC: Area under the curve
ER: Estrogen receptor
BMI: Body mass index
